# Dynapenia and sarcopenia: association with the diagnosis, duration and complication of type 2 diabetes mellitus in ELSA-Brasil

**DOI:** 10.1590/0102-311XEN081223

**Published:** 2024-02-02

**Authors:** Débora Noara Duarte dos Santos, Carolina Gomes Coelho, Maria de Fátima Haueisen Sander Diniz, Bruce Bartholow Duncan, Maria Inês Schmidt, Isabela Judith Martins Bensenor, Claudia Szlejf, Rosa Weiss Telles, Sandhi Maria Barreto

**Affiliations:** 1 Faculdade de Medicina, Universidade Federal de Minas Gerais, Belo Horizonte, Brasil.; 2 Hospital das Clínicas da Universidade Federal de Minas Gerais, Belo Horizonte, Brasil.; 3 Programa de Pós-graduação em Epidemiologia, Universidade Federal do Rio Grande do Sul, Porto Alegre, Brasil.; 4 Hospital de Clínicas de Porto Alegre, Universidade Federal do Rio Grande do Sul, Porto Alegre, Brasil.; 5 Universidade de São Paulo, São Paulo, Brasil.; 6 Hospital Universitário, Universidade de São Paulo, São Paulo, Brasil.

**Keywords:** Sarcopenia, Hand Grip Strength, Muscle Strength, Type 2 Diabetes Mellitus, Sarcopenia, Força da Mão, Força Muscular, Diabetes Mellitus Tipo 2, Sarcopenia, Fuerza de la Mano, Fuerza Muscular, Diabetes Mellitus Tipo 2

## Abstract

Sarcopenia (the loss of muscle mass, strength and skeletal muscle function) increases mortality and the risk of hospitalization in the older population. Although it is known that older adults with type 2 diabetes mellitus (T2DM) have a higher risk of dynapenia and sarcopenia, few studies have investigated these conditions in middle-aged populations. The objective of this study was to investigate whether T2DM, its duration, the presence of albuminuria, and glycemic control are associated with sarcopenia and its components in adults. The cross-sectional analysis was based on data from visit 2 of the *Brazilian Longitudinal Study of Adult Health* (2012-2014). The 2018 European Working Group on Sarcopenia in Older People criteria were used to define dynapenia, low appendicular muscle mass (LAMM), and sarcopenia (absent/probable/confirmed). The explanatory variables were: T2DM; duration of T2DM; T2DM according to the presence of albuminuria; and glycemic control (HbA1C < 7%) among people with T2DM. A total of 12,132 participants (mean age = 55.5, SD: 8.9 years) were included. The odds ratio for LAMM was greater among those with T2DM, T2DM duration from 5 to 10 years, and T2DM without albuminuria. Chances of dynapenia were higher among those with T2DM, T2DM duration > 10 years, and T2DM with and without albuminuria. The variables T2DM, T2DM ≥ 10 years, and T2DM with albuminuria increased the odds of probable sarcopenia, and T2DM duration from 5 to 10 years increased the odds of confirmed sarcopenia. The results support the importance of frequently monitoring the musculoskeletal mass and strength of individuals with T2DM to prevent sarcopenia and related outcomes.

## Resumo

A sarcopenia (perda de massa muscular, força e função muscular esquelética) aumenta a mortalidade e o risco de hospitalização em idosos. Idosos com diabetes mellitus tipo 2 (DMT2) apresentam risco elevado de desenvolver dinapenia e sarcopenia, mas poucos estudos investigaram populações de meia-idade. O objetivo foi investigar se DMT2, sua duração, a presença de albuminúria e o controle glicêmico estão associados à sarcopenia e seus componentes em adultos. Análise transversal baseada nos dados da segunda visita do *Estudo Longitudinal de Saúde do Adulto* (2012-2014). Os critérios do European Working Group on Sarcopenia in Older People [Grupo de Trabalho Europeu sobre Sarcopenia em Pessoas Idosas] de 2018 foram usados para definir dinapenia, baixa massa muscular apendicular e sarcopenia (ausente/provável/confirmada). As variáveis explicativas foram: DMT2; duração do DMT2; DMT2 de acordo com a presença de albuminúria; e controle glicêmico (HbA1c < 7%) entre pessoas com DMT2. Foram incluídos 12.132 participantes (idade média de 55,5; DP: 8,9 anos). A razão de chances para baixa massa muscular apendicular foi maior entre pessoas com DMT2, duração do DMT2 entre 5 e 10 anos e DMT2 sem albuminúria. As chances de dinapenia foram maiores entre pessoas com DMT2, duração do DMT2 > 10 anos e DMT2 com e sem albuminúria. DMT2, DMT2 ≥ 10 anos e DMT2 com albuminúria aumentaram as chances de sarcopenia provável e duração do DMT2 entre 5 e 10 anos aumentaram as chances de sarcopenia confirmada. Os resultados reforçam a importância do monitoramento frequente da massa e da força muscular em indivíduos com DMT2 para prevenir a sarcopenia e desfechos relacionados.

## Resumen

La sarcopenia (pérdida de masa muscular, fuerza y función muscular esquelética) aumenta la mortalidad y el riesgo de hospitalización en ancianos. Los ancianos con diabetes mellitus tipo 2 (DMT2) presentan un mayor riesgo de sufrir dinapenia y sarcopenia, pero pocos estudios han investigado poblaciones de mediana edad. El objetivo fue investigar si la DMT2, su duración, la presencia de albuminuria y el control glucémico están asociados con la sarcopenia y sus componentes en adultos. Análisis transversal basado en los datos de la visita 2 del *Estudio Longitudinal de Salud del Adulto en Brasil* (2012-2014). Se utilizaron los criterios del European Working Group on Sarcopenia in Older People [Grupo de Trabajo Europeo sobre Sarcopenia en Personas Mayores] del 2018 para definir dinapenia, baja masa muscular apendicular y sarcopenia (ausente/probable/confirmada). Las variables explicativas fueron las siguientes: DMT2; duración de la DMT2; DMT2 según la presencia de albuminuria; y control glucémico (HbA1c < 7%) entre personas con DMT2. Se incluyeron 12.132 participantes (edad media = 55,5, DE: 8,9 años). La razón de probabilidades de masa muscular apendicular baja fue mayor entre personas con DMT2, duración de la DMT2 entre 5 y 10 años y DMT2 sin albuminuria. Las probabilidades de dinapenia fueron mayores entre las personas con DMT2, duración de la DMT2 > 10 años y DMT2 con y sin albuminuria. Las condiciones de DMT2, DMT2 ≥ 10 años y DMT2 con albuminuria aumentaron las probabilidades de sarcopenia probable y la duración de la DMT2 entre 5 y 10 años las probabilidades de sarcopenia confirmada. Los resultados refuerzan la importancia del monitoreo frecuente de la masa y de la fuerza musculoesquelética en individuos con DMT2 para prevenir la sarcopenia y los desenlaces relacionados.

## Introduction

Sarcopenia is a multifactorial muscle disease characterized by loss of strength and muscle mass ^1^ ^,^ ^2^. It is a major public health problem that causes loss of independence and high morbidity and mortality; in addition, it has high personal and social health care costs, due to frequent hospital admissions and increased institutionalization ^3^. For a long time, sarcopenia was solely related to aging and was studied primarily in the older adults ^4^. Its definition underwent successive changes until 2018, when the European Working Group on Sarcopenia in Older People (EWGSOP2) started to consider low muscle strength (dynapenia) the first parameter that should be assessed in individuals with suspected sarcopenia, as it is the most reliable measure of muscle function ^1^.

The prevalence of sarcopenia varies greatly worldwide, ranging from 1%-29% in community-dwelling individuals aged over 50 years to 14%-33% among those living in long-term care facilities ^5^. A case-control study on chronic kidney disease based on the UK Biobank (United Kingdom) found a prevalence of 0.2% of confirmed sarcopenia in its control group, composed by adults (mean age = 56.1, standard deviation - SD: 8.1 years), using the EWGSOP2 criteria ^6^. The large variation in sarcopenia prevalence may be caused by factors such as diagnostic criteria and the sociodemographic and health characteristics of the populations studied, especially age range and the prevalence of comorbidities ^7^ ^,^ ^8^ ^,^ ^9^. In Brazil, which is a middle-income country with fast-ageing population, a systematic review of 31 studies identified that the prevalence of sarcopenia (defined as low muscle mass, low muscle function, or low muscle mass plus low muscle function) is 16% among individuals aged 60 years or older ^10^. Low muscle mass levels were identified using magnetic resonance imaging, computed tomography, dual-energy X-ray absorptiometry (DEXA), and bioelectrical impedance or anthropometry; and low muscle function levels were defined by low hand grip and/or knee extension/flexion strength, or low physical performance according to components of the Short Physical Performance Battery and the Timed Up & Go test ^10^.

The prevalence of dynapenia also varies according to different definitions and scenarios (e.g., health conditions and characteristics of individuals), ranging from 17.8% in the European population aged 70 or over ^11^ to 71.2% in a Falls and Fractures Clinic in Colombia ^12^. The *Brazilian Longitudinal Study of Aging* (ELSI-Brazil) revealed a high prevalence of dynapenia (17.2%) in older adults ^13^.

Type 2 diabetes mellitus (T2DM) is a chronic noncommunicable metabolic disease with important systemic repercussions on the macro and microvasculature of individuals ^14^. Recent studies have shown that people with T2DM tend to have a significantly higher impairment of muscle function than individuals without the disease ^1^ ^,^ ^3^ ^,^ ^9^. Moreover, T2DM may also contribute to accelerating dynapenia progression and cause significant degradation of lean mass, alone or associated with low muscle strength, even in adults ^1^ ^,^ ^14^ ^,^ ^15^ ^,^ ^16^ ^,^ ^17^.

Even with the aforementioned data, critical gaps remain in the literature on the relationship between T2DM and sarcopenia. The use of different definitions and cut-off points to define sarcopenia, the recent changes in dynapenia and sarcopenia parameters, and the lack of studies including middle-aged adults limit conclusions about the relationship between T2DM and sarcopenia. The association of sarcopenia with T2DM-specific characteristics such as duration of diagnosis, disease complications, and glycemic control have been scarcely studied. Given this scenario, this study sought to investigate the association of T2DM and its factors, including the duration of diagnosis, presence of albuminuria, and glycemic control, with sarcopenia and each sarcopenia component in Brazilian middle-aged and older adults. We hypothesize that T2DM is associated with greater chances of sarcopenia and that the magnitude of the associations is greater among individuals with a longer duration of diagnosis, those with albuminuria, and those with inadequate glycemic control.

## Materials and methods

### Study design and population

This is a cross-sectional study that used data from the visit 2 of the *Brazilian Longitudinal Study of Adult Health* (ELSA-Brasil), a cohort consisting, at baseline (2008-2010), of 15,105 active and retired civil servants aged 35-74 years who worked in public higher education institutions and research centers in six Brazilian states: Minas Gerais, São Paulo, Rio de Janeiro, Rio Grande do Sul, Espírito Santo, and Bahia.

Muscle strength and body composition examinations began to be conducted in the visit 2 of the ELSA-Brasil cohort (2012-2014), which included 14,014 individuals. All participants with valid information regarding T2DM, handgrip strength (HGS), and bioelectrical impedance analysis (BIA) were included (n = 12,478). In order to avoid including individuals with type 1 diabetes mellitus, this study excluded participants who reported, at baseline of ELSA-Brasil, that they had been medically diagnosed with diabetes before 30 years of age (n = 45) and/or used insulin as the first medication for the treatment of diabetes (n = 32). In addition, this study excluded individuals with a self-reported medical diagnosis of stroke (n = 131), due to the well-defined association of this morbidity with sarcopenia, and those who, in visit 2, reported having undergone bariatric surgery (n = 138), due to the possibility of remission of the T2DM diagnosis during the study follow-up period. Considering these overlapping criteria, the final analytic sample consisted of 12,132 participants.

ELSA-Brasil was approved by the Research Ethics Committee of each participating institution under registries 669/06 (São Paulo University), 343/06 (Oswaldo Cruz Foundation), 041/06 (Espírito Santo Federal University), 186/06 (Minas Gerais Federal University), 194/06 (Rio Grande do Sul Federal University), and 027/06 (Bahia Federal University). All participants were informed about the objectives of the study and signed an informed consent form.

### Procedures

In ELSA-Brasil, data collection was conducted via face-to-face interviews, clinical and laboratory examinations, and anthropometric measurements. Trained and certified professionals performed all measurements and evaluations according to pre-established protocols ^18^.

#### Investigation of sarcopenia and its components, and definition of the outcome variables

Muscle strength was investigated using HGS, which was obtained using a Jamar hydraulic hand dynamometer (Sammons Preston, https://www.performancehealth.com/products/brand/sammons-preston). Three measurements were made on each hand, with an interval of approximately one minute between them. The measure with the highest value in kilograms (kg) was used to define HGS.

Skeletal muscle mass was obtained using a vertical tetrapolar BIA device, which uses 8-point tactile electrodes (Inbody230; InBody Co., https://inbody.com). For the test, all participants were instructed not to exercise during the 24 hours prior to the test, not to drink caffeine or alcohol 12 hours before the test, and to empty their bladder just before the test. The BIA result is presented as a percentage of total, appendicular and trunk lean mass, adjusted for sex and age. The present study used appendicular muscle mass adjusted by height squared ^1^.

Sarcopenia and its components were defined according to the revised EWGSOP2 criteria ^1^, as follows: presence of low muscle strength (dynapenia), when HGS was < 27kg for men and < 16kg for women; low appendicular muscle mass (LAMM), when appendicular muscle mass corrected for squared height was < 7kg/m^2^ for men and < 5.5kg/m^2^ for women; and sarcopenia was determined by the simultaneous presence of dynapenia and LAMM.

Based on these criteria, three outcomes were analyzed separately: (a) LAMM (yes/no); (b) dynapenia (yes/no); and (c) sarcopenia, categorized as absent (reference), probable, when only dynapenia was present, and confirmed, when both LAMM and dynapenia were present. Individuals with isolated LAMM were considered to have no sarcopenia and included in the reference category, in accordance with the EWGSOP2.

#### T2DM and related variables

The presence of T2DM (yes/no) was investigated in all participants included in the present study with two means (questionnaire and laboratory measures), and defined by a self-reported diagnosis and/or the presence of at least one altered laboratory measurement, namely: fasting blood glucose ≥ 126mg/dL and/or blood glucose 2 hours after dextrosol ≥ 200mg/dL and/or glycated hemoglobin (HbA1c) ≥ 6.5 ^19^ ^,^ ^20^ ^,^ ^21^. The self-reported diagnosis of T2DM was obtained by a positive response to one of the following questions: “Has a physician ever informed you that you have or had diabetes?” and “Have you used diabetes medication in the past two weeks?”, and self-reported T2DM criteria were met by 1,371 (61.9%) of the participants with diabetes.

Blood was drawn from the participants after they underwent a 12 hours fast. Plasma glucose was measured using the hexokinase enzyme method (ADVIA Chemistry; Siemens, https://www.siemens-healthineers.com); HbA1c was measured with high-pressure liquid chromatography (Bio-RadLaboratories, https://www.bio-rad.com/), using a method certified by the U.S. National Glycohemoglobin Standardization Program ^18^.

The duration of the T2DM diagnosis was obtained by subtracting the age of T2DM diagnosis from the age of the individual during visit 2 of the ELSA-Brasil, then divided into four categories: no T2DM (reference category), T2DM < 5 years, T2DM between 5 and 10 years, and T2DM ≥ 10 years.

The presence of albuminuria (yes/no) was defined by a urinary albumin/creatinine ratio (ACR) greater than or equal to 30mg/g in visit 2 ^22^ ^,^ ^23^. Urinary creatinine was measured with the colorimetric enzymatic assay (Jaffé method, ADVIA Chemistry) and urinary albumin was measured using the immunochemical assay (nephelometry; Dade Behring, https://www.siemens.com/), both applied to 12-hour urine overnight samples ^18^. The T2DM variable was categorized into three groups according to the presence of albuminuria: absence of T2DM (reference), T2DM without albuminuria (ACR < 30mg/g), and T2DM with albuminuria (ACR ≥ 30mg/g). Individuals with ACR ≥ 30mg/g and no T2DM remained in the reference category.

Lastly, the glycemic control analysis was restricted to participants who reported having already been diagnosed with T2DM (prevalent cases), i.e., individuals without T2DM or with T2DM identified only by laboratory findings during visit 2 of the ELSA-Brasil were not included in this analysis. The HbA1c test was used to identify T2DM glycemic control: individuals with HbA1c < 7% (reference category) were considered to have controlled T2DM and individuals with HbA1c ≥ 7% were considered to have uncontrolled T2DM ^24^.

#### Covariates

Sociodemographic, behavioral, and health indicators were included as covariate variables in the statistical models. The sociodemographic data were: sex; age (continuous scale); education level (university degree, high school, complete elementary school or incomplete elementary school); marital status (married/cohabiting, separated/divorced, widowed, single or other); and race/color (black, brown, white, yellow or Indigenous).

Health-related behaviors included leisure-time physical activity (low, moderate or vigorous), which was assessed using the *International Physical Activity Questionnaire* (IPAQ) ^25^, excessive alcohol consumption (yes/no), assessed with the semi-quantitative *Food Frequency Questionnaire* (SQ-FFQ) and defined as ≥ 210g of alcohol per week for men and ≥ 140g per week for women ^25^, and smoking (never smoked/ex-smoker/smoker). The body mass index (BMI) was calculated from body weight divided by height squared and used on a continuous scale.

Self-reported cardiovascular disease (CVD) (yes/no) included the following conditions: acute myocardial infarction; angina; congestive heart failure; and coronary artery bypass grafting. Renal dysfunction was defined by estimated glomerular filtration rate (eGFR) < 60mL/minute/1.73m^2^, and eGFR was estimated with the Chronic Kidney Disease Epidemiology Collaboration (CKD-EPI) equation, without adjustment for race/color ^23^.

The use of continuous medication in the last two weeks was assessed during an interview and by checking the packaging, package inserts, and/or cartridges of the medication used. The use of angiotensin-converting enzyme inhibitors (ACEI) and/or angiotensin receptor blockers (ARB) in visit 2 was considered for adjustment.

Finally, the self-reported medical diagnosis of rheumatic diseases and cancer in visit 1, the use of oral or parenteral corticosteroids, and the use of drugs for the treatment of rheumatoid arthritis and systemic lupus erythematosus (abatacepte, adalimumab, azathioprine, cyclosporine, hydroxychloroquine, leflunomide, methotrexate, sulfasalazine) were assessed.

### Statistical analysis

Descriptive analyses were conducted using mean and SD (for continuous variables) and frequencies and percentages (for categorical variables). Differences between groups were investigated with the chi-square test. The association of T2DM, the variables related to T2DM, and the components of sarcopenia, LAMM (yes/no) and dynapenia (yes/no), was analyzed using a logistic regression model. The association of these variables with sarcopenia, which was identified according to the EWGSOP2 (absent/probable/confirmed), was assessed using multinomial logistic regression models.

All analyses followed the steps described as follows, and missing values were removed from the final models. We first investigated the association between the presence of T2DM and each response variable. When the final model showed a statistical association (p < 0.05) between T2DM and the response variable in question, the associations with the other T2DM-related explanatory variables (duration of diagnosis, presence of albuminuria, and glycemic control of T2DM) were investigated.

After the bivariate regression analyses (crude model) were conducted, sequential adjustments were made with the following covariates: BMI (model 1); sex, age, education level, marital status, and race/color (model 2); physical activity, excessive alcohol consumption, and smoking (model 3); CVD and chronic renal failure (model 4 - adjusted model). Additionally, the variable HbA1c (continuous) was added to the fully adjusted models with T2DM as an explanatory variable to evaluate if the association obtained was independent of the glycemic levels.

The variable use of ACEI and/or ARBs (yes/no) in visit 2 was added to the final models investigating the association between T2DM according to the presence of albuminuria and the outcome variables. Sensitivity analyses were also performed, excluding participants who reported using oral/parenteral corticosteroids, and those who had self-reported diagnoses of cancer and rheumatic diseases (with the concurrent use of specific medications). Lastly, interactions between T2DM and age were investigated (due to the already known association of these variables), and were not significant (data not shown).

The magnitude of the associations found was estimated using the odds ratio (OR), with a 95% confidence interval (95%CI). For all logistic regression models, the Hosmer-Lemeshow goodness-of-fit test was performed.

The data were analyzed using the Stata software, version 14.0 (https://www.stata.com), with a 95% confidence level.

## Results

Most of the 12,132 participants were female (54.3%). The sample had a mean age of 55.5 (SD: 8.9) years (total age ranging from 38 to 79 years), and 18.3% of participants had T2DM. The overall prevalence of LAMM was 6.1% and that of dynapenia was 3.9%, with dynapenia being more frequent in women (4.5%). The prevalence of both LAMM and dynapenia rose in parallel with decreases in educational levels and with increases in age (Table 1). Regarding sarcopenia, defined according to the EWGSOP2, the frequency of probable sarcopenia was higher in women (3.8%) and rose in parallel with increases in age and with decreases in educational levels (Table 1).

**Table 1:** Description of sociodemographic and health characteristics of participants in the visit 2 of *Brazilian Longitudinal Study of Adult Health* (ELSA-Brasil), 2012-2014 (n = 12,132).

Characteristics	Total (%) (n = 12,132)	Prevalence of individual components of sarcopenia		Prevalence of sarcopenia according to the EWGSOP2	
		LAMM * (n = 744; 6.13%)	Dynapenia ** (n = 472; 3.89%)	Probable sarcopenia ** (n = 379; 3.12%)	Confirmed sarcopenia *** (n = 93; 0.77%)
Sex					
Male	45.71	6.44	3.12	2.34	0.78
Female	54.29	5.88	4.54	3.78	0.76
p-value		0.200	< 0.001	< 0.001	
Age (years)					
35-44	10.54	2.11	2.19	2.11	0.08
45-54	38.30	3.59	2.52	2.22	0.30
55-64	34.08	6.38	3.80	3.05	0.75
65-74	14.59	12.03	6.84	5.20	1.64
> 75	2.48	24.25	16.28	10.30	5.98
p-value		< 0.001	< 0.001	< 0.001	
Education ^#^					
Higher education	58.19	5.75	3.70	3.05	0.65
High school	30.75	5.69	3.35	2.74	0.62
Complete elementary school	6.08	7.06	5.83	4.61	1.22
Incomplete elementary school	4.98	12.09	7.12	4.64	2.48
p-value		< 0.001	< 0.001	< 0.001	
Marital status ^##^					
Married/Cohabiting	64.81	5.62	3.41	2.80	0.61
Separated/Divorced	15.98	5.73	4.08	3.25	0.83
Widowed	4.82	8.73	6.85	5.14	1.71
Single	13.60	8.07	4.85	3.70	1.15
Other	0.79	0.81	5.21	5.21	0.00
p-value		< 0.001	< 0.001	0.001	
Race/Color ^###^					
Black	14.80	4.35	4.32	3.48	0.85
Brown	26.86	6.47	3.53	2.58	0.95
White	53.56	6.11	2.73	2.51	0.22
Yellow and Indigenous	3.67	11.91	4.94	4.27	0.67
p-value		< 0.001	0.007	0.004	
Physical activity ^§^					
Low	73.88	6.41	4.10	3.29	0.80
Moderate	18.05	5.90	3.80	3.02	0.78
Vigorous	8.07	3.99	2.15	1.74	0.41
p-value		0.010	0.011	0.059	
Excessive alcohol consumption ^§§^					
No	91.93	6.17	3.98	3.19	0.78
Yes	8.07	5.52	2.97	2.35	0.61
p-value		0.414	0.117	0.291	
Smoking ^§§§^					
Never smoked	58.31	6.48	3.89	3.03	0.86
Ex-smoker	30.60	5.04	3.91	3.37	0.54
Current smoker	11.09	7.29	3.79	2.90	0.89
p-value		0.002	0.982	0.311	
BMI (kg/m^2^) [mean (SD)] *	27.53 (4.85)	21.91 (2.63)	27.14 (4.96)	28.24 (4.73)	22.66 (2.98)
Cardiovascular disease ^†,††^					
Absent	96.78	6.09	3.75	3.00	0.75
Present	3.22	7.18	7.69	6.67	1.03
p-value		0.378	< 0.001	< 0.001	
Renal insufficiency ^†††,‡^					
Absent	93.95	5.98	3.69	3.00	0.69
Present	6.05	8.45	6.95	5.04	1.91
p-value		0.007	< 0.001	< 0.001	
T2DM					
Absent	81.75	6.26	3.47	2.77	0.70
Present	18.25	5.56	5.78	4.70	1.08
p-value				0.211	< 0.001
Duration of T2DM (years)					
Without T2DM	81.75	6.26	3.47	2.77	0.70
< 5	11.34	4.72	4.72	4.14	0.58
≥ 5 to < 10	3.54	5.81	5.58	4.19	1.40
≥ 10	3.36	8.09	9.56	7.11	2.45
p-value		0.050	< 0.001	< 0.001	
T2DM according to the presence of albuminuria ^‡‡,‡‡‡^					
Without T2DM	81.82	6.31	3.42	2.74	0.68
T2DM without albuminuria	15.62	5.37	5.20	4.38	0.82
T2DM with albuminuria	2.57	5.67	9.33	7.00	2.33
p-value		0.285	< 0.001	< 0.001	
Glycemic control in T2DM ^‖^					
Controlled	60.51	6.40	7.05	5.09	1.96
Uncontrolled	39.49	5.20	5.00	4.20	0.80
p-value		0.378	0.141	0.188	
HbA1C (%) [mean (SD)]	5,46 (0,98)	5,43 (0,96)	5,56 (1,05)	5,58 (1,09)	5,49 (0,86)

BMI: body mass index ; EWGSOP2: European Working Group on Sarcopenia in Older People Consensus 2; HbA1c: glycated hemoglobin; LAMM: low appendicular muscle mass; SD: standard deviation; T2DM: type 2 diabetes mellitus.

* < 7.0kg/m^2^ in men and < 5.5kg/m^2^ in women;

** Handgrip strength in men < 27kg and in women < 16kg;

*** Simultaneous presence of dynapenia and LAMM;

^#^ Missing data: 6 participants;

^##^ Missing data: 7 participants;

^###^ Missing data: 135 participants;

^§^ Missing data: 13 participants;

^§§^ Missing data: 11 participants;

^§§§^ Missing data: 8 participants;

^†^ Acute myocardial infarction, angina, congestive heart failure, and coronary artery bypass grafting;

^††^ Missing data: 19 participants;

^†††^ According to glomerular filtration rate (GFR) calculated by Chronic Kidney Disease Epidemiology Collaboration (CKD-EPI) formula. Absent: GFR ≥ 60mL/minute; present: GFR < 60mL/minute;

^‡^ Missing data: 2 participants;

^‡‡^ According to the urinary albumin/creatinine ratio. T2DM without albuminuria: albuminuria < 30mg/g; T2DM with albuminuria: albuminuria ≥ 30mg/g;

^‡‡‡^ Missing data: 441 participants;

^‖^ According to HbA1c, in individuals with T2DM prior to visit 2. Controlled: HbA1c < 7%; uncontrolled: HbA1c ≥ 7%.

Furthermore, the prevalences of probable and confirmed sarcopenia were higher among individuals with T2DM - especially those who had T2DM with albuminuria - and increased progressively with a longer duration of T2DM diagnosis (Table 1).

T2DM increased the odds of LAMM (OR = 1.51; IC95%: 1.12-2.04) after all adjustments for confounding factors (Table 2). For the duration of T2DM, a positive association with LAMM was identified only in individuals diagnosed between 5 and 10 years (OR = 2.14; 95%CI: 1.30-3.53) (Table 3). The presence of albuminuria and glycemic control in T2DM was not statistically associated with LAMM (Table 3).

**Table 2:** Association between diagnosis of type 2 diabetes mellitus (T2DM) and components of dynapenia and sarcopenia according to the European Working Group on Sarcopenia in Older People Consensus 2 (EWGSOP2). *Brazilian Longitudinal Study of Adult Health* (ELSA-Brasil), 2012-2014 (n = 12,132).

T2DM	Individual components of sarcopenia (logistic regression models)				Sarcopenia according to EWGSOP2 (multinomial regression models)			
	LAMM *		Dynapenia **		Crude model OR (95%CI)		Adjusted model *** OR (95%CI)	
	Crude model OR (95%CI)	Adjusted model *** OR (95%CI)	Crude model OR (95%CI)	Adjusted model *** OR (95%CI)	Probable sarcopenia **	Confirmed sarcopenia ^#^	Probable sarcopenia **	Confirmed sarcopenia ^#^
Absent	1.00	1.00	1.00	1.00	1.00	1.00	1.00	1.00
Present	0.88 (0.72-1.08)	1.51 (1.12-2.04) ^##^	1.71 (1.39-2.10) ^###^	1.54 (1.18-2.01) ^##^	1.74 (1.38-2.19) ^##^	1.60 (1.00-2.55)	1.43 (1.07-1.93) ^##^	2.50 (1.39-4.50) ^##^

95%CI: 95% confidence interval; LAMM: low appendicular muscle mass; OR: odds ratio.

* < 7.0kg/m^2^ in men and < 5.5kg/m^2^ in women;

** Handgrip strength in men < 27kg and in women < 16kg;

*** Adjusted for body mass index (continuous); age; education (higher education, high school, complete elementary school, incomplete elementary school); marital status: married/cohabiting, separated/divorced, widowed, single, other); race/color (black, brown, white, yellow and Indigenous); physical activity (low, moderate or vigorous); excessive alcohol consumption (yes/no); smoking (never smoked, ex-smoker, current smoker); cardiovascular diseases (absent/present), renal dysfunction (absent/present); and glycated hemoglobin (continuous);

^#^ Simultaneous presence of dynapenia and LAMM;

^##^ p < 0.05;

^###^ p<0.001.

**Table 3:** Association between duration of type 2 diabetes mellitus (T2DM), T2DM according to the presence of albuminuria, and glycemic control in T2DM with low appendicular muscle mass (LAMM). *Brazilian Longitudinal Study of Adult Health* (ELSA-Brasil), 2012-2014 (n = 12,132).

LAMM *	Crude model OR (95%CI)	Adjusted model ** OR (95%CI)
Duration of T2DM (years)		
Without	1.00	1.00
< 5	0.74 (0.57-0.97) ***	1.27 (0.92-1.77)
≥ 5 to < 10	0.92 (0.61-1.40)	2.14 (1.30-3.53) ***
≥ 10	1.32 (0.92-1.90)	1.44 (0.91-2.30)
T2DM according to the presence of albuminuria ^#^		
Without T2DM	1.00	1.00
T2DM without albuminuria	0.84 (0.68-1.05)	1.43 (1.08-1.89) ***
T2DM with albuminuria	0.89 (0.54-1.46)	1.25 (0.67-2.33)
Glycemic control in T2DM ^##^		
Controlled	1.00	1.00
Uncontrolled	0.80 (0.49-1.31)	0.90 (0.47-1.72)

95%CI: 95% confidence interval; OR: odds ratio.

* < 7.0kg/m^2^ in men and < 5.5kg/m^2^ in women;

** Adjusted for body mass index (continuous); age; education (higher education, high school, complete elementary school, incomplete elementary school); marital status: married/cohabiting, separated/divorced, widowed, single, other); race/color (black, brown, white, yellow and Indigenous); physical activity (low, moderate or vigorous); excessive alcohol consumption (yes/no); smoking (never smoked, ex-smoker, current smoker); cardiovascular diseases (absent/present); and renal dysfunction (absent/present);

*** p < 0.05;

^#^ T2DM without albuminuria: albuminuria/creatinine ratio < 30mg/g; T2DM with albuminuria: albuminuria/creatinine ratio ≥ 30mg/g;

^##^ Only in individuals with T2DM prior to visit 2. Controlled: HbA1c < 7%; uncontrolled: HbA1c ≥ 7%.

T2DM also increased the odds of dynapenia (OR = 1.54; IC95%: 1.18-2.01) (Table 2). Regarding the duration of T2DM, only T2DM cases that lasted > 10 years remained statistically associated with dynapenia after adjustments (OR = 2.00; 95%CI: 1.38-2.92) (Table 4). In fully adjusted models, both T2DM without (OR = 1.31; 95%CI: 1.02-1.68) and with albuminuria (OR = 2.22; 95%CI: 1.44-3.43) were associated with higher odds of dynapenia, and the magnitude of association was greater in the latter (Table 4). In individuals with T2DM, glycemic control was not statistically associated with dynapenia (Table 4).

**Table 4:** Association between duration of type 2 diabetes mellitus (T2DM), T2DM according to the presence of albuminuria, and glycemic control in T2DM with dynapenia. *Brazilian Longitudinal Study of Adult Health* (ELSA-Brasil), 2012-2014 (n = 12,132).

Dynapenia *	Crude model OR (95%CI)	Adjusted model ** OR (95%CI)
Duration of T2DM (years)		
Without	1.00	1.00
< 5	1.38 (1.05-1.81) ***	1.22 (0.91-1.62)
≥ 5 to < 10	1.65 (1.08-2.52) ***	1.31 (0.84-2.03)
≥ 10	2.94 (2.08-4.16) ^#^	2.00 (1.38-2.92) ^#^
T2DM according to the presence of albuminuria ^##^		
Without T2DM	1.00	1.00
T2DM without albuminuria	1.55 (1.23-1.96) **	1.31 (1.02-1.68) ***
T2DM with albuminuria	2.91 (1.94-4.36) **	2.22 (1.44-3.43) ***
Glycemic control in T2DM ^###^		
Controlled	1.00	1.00
Uncontrolled	0.69 (0.43-1.13)	0.77 (0.41-1.30)

95%CI: 95% confidence interval; OR: odds ratio.

* Handgrip strength in men < 27kg and in women < 16kg;

** Adjusted for body mass index (continuous); age; education (higher education, high school, complete elementary school, incomplete elementary school); marital status: married/cohabiting, separated/divorced, widowed, single, other); race/color (black, brown, white, yellow and Indigenous); physical activity (low, moderate or vigorous); excessive alcohol consumption (yes/no); smoking (never smoked, ex-smoker, current smoker); cardiovascular diseases (absent/present); and renal dysfunction (absent/present);

*** p < 0.05;

^#^ p < 0.001;

^##^ T2DM without albuminuria: albuminuria/creatinine ratio < 30mg/g; T2DM with albuminuria: albuminuria/creatinine ratio ≥ 30mg/g;

^###^ Only in individuals with T2DM prior to visit 2. Controlled: HbA1c < 7%; uncontrolled: HbA1c ≥ 7%.

After all the adjustments, it was found that T2DM increased the chances of probable sarcopenia by about 40% (OR = 1.43; 95%CI: 1.07-1.93) and the chances of confirmed sarcopenia by more than double (OR = 2.50; 95%CI: 1.39-4.50) (Table 2). Regarding the duration of T2DM diagnosis, only a duration ≥ 10 years (OR = 1.75; 95%CI: 1.21-2.84) increased the odds of probable sarcopenia, while a duration of ≥ 5 to < 10 years increased the odds of confirmed sarcopenia by 3.30-fold (95%CI: 1.32-8,27) and a duration ≥ 10 years increased the odds by 3.74-fold (95%CI: 1.77-7.55) (Table 5). Both T2DM without and with albuminuria were associated with probable (OR = 1.27; 95%CI: 0.97-1.68 and OR = 2.04; 95%CI: 1.25-3.32, respectively) and confirmed sarcopenia (OR = 1.76; 95%CI: 0.96-3.22 and OR = 3.81; 95%CI: 1.50-9.67, respectively) (Table 5), even though the association of T2DM without albuminuria with confirmed sarcopenia was on the borderline of statistical significance. No statistical association was found between T2DM glycemic control and probable or confirmed sarcopenia among individuals with prevalent T2DM (Table 5).

**Table 5:** Association between duration of type 2 diabetes mellitus (T2DM), T2DM according to the presence of albuminuria, and glycemic control in T2DM with sarcopenia according to the European Working Group on Sarcopenia in Older People Consensus 2 (EWGSOP2). *Brazilian Longitudinal Study of Adult Health* (ELSA-Brasil), 2012-2014 (n = 12,132).

Sarcopenia (EWGSOP2)	Crude model OR (95%CI)		Adjusted model * OR (95%CI)	
	Probable sarcopenia **	Confirmed sarcopenia ***	Probable sarcopenia **	Confirmed sarcopenia ***
Duration of T2DM (years)				
Without	1.00	1.00	1.00	1.00
< 5	1.51 (1.13-2.02) ^#^	0.85 (0.41-1.77)	1.22 (0.90-1.67)	1.11 (0.51-2.42)
≥ 5 to < 10	1.54 (0.95-2.51)	2.05 (0.89-4.75)	1.14 (0.69-1.89)	3.30 (1.32-8.27) ^#^
≥ 10	2.74 (1.84-4.07) ^##^	3.76 (1.92-7.36) ^##^	1.75 (1.21-2.84) ^#^	3.74 (1.77-7.55) ^#^
T2DM according to the presence of albuminuria ^###^				
Without T2DM	1.00	1.00	1.00	1.00
T2DM without albuminuria	1.63 (1.26-2.10) ^##^	1.23 (0.70-2.16)	1.27 (0.97-1.68)	1.76 (0.96-3.22)
T2DM with albuminuria	2.72 (1.72-4.32) ^##^	3.66 (1.66-8.05) ^#^	2.04 (1.25-3.32) ^#^	3.81 (1.50-9.67) ^#^
Glycemic control in T2DM ^§^				
Controlled	1.00	1.00	1.00	1.00
Uncontrolled	0.81 (0.47-1.39)	0.40 (0.13-1.21)	0.89 (0.50-1.59)	0.50 (0.15-1.70)

95%CI: 95% confidence interval; OR: odds ratio.

* Adjusted for body mass index (continuous); age; education (higher education, high school, complete elementary school, incomplete elementary school); marital status: married/cohabiting, separated/divorced, widowed, single, other); race/color (black, brown, white, yellow and Indigenous); physical activity (low, moderate or vigorous); excessive alcohol consumption (yes/no); smoking (never smoked, ex-smoker, current smoker); cardiovascular diseases (absent/present); and renal dysfunction (absent/present);

** Handgrip strength in men < 27kg and in women < 16kg;

*** Simultaneous presence of dynapenia and low appendicular muscle mass;

^#^ p < 0.05;

^##^ p < 0.001;

^###^ T2DM without albuminuria: albuminuria/creatinine ratio < 30mg/g; T2DM with albuminuria: albuminuria/creatinine ratio ≥ 30mg/g;

^§^ Only in individuals with T2DM prior to visit 2. Controlled: HbA1c < 7%; uncontrolled: HbA1c ≥ 7%.

Including the adjustment for ACEI and ARB use in the final models investigating the association between T2DM with and without albuminuria and response variables hardly changed the OR (Tables 6, 7, and 8). Sensitivity analyses excluding individuals with cancer, rheumatic diseases, and those who used oral/parenteral corticosteroid showed no changes in the directions of the associations between the presence of T2DM and the response variables and no major changes in the magnitude of the associations (Tables 9, 10, and 11).

**Table 6:** Association between type 2 diabetes mellitus (T2DM) according to the presence of albuminuria and low appendicular muscle mass (LAMM), with further adjustment for the use of angiotensin-converting enzyme inhibitor drugs and angiotensin receptor blockers. *Brazilian Longitudinal Study of Adult Health* (ELSA-Brasil), 2012-2014 (n = 11,658).

LAMM *	Crude model OR (95%CI) ***	Adjusted model ** OR (95%CI) ***
T2DM according to the presence of albuminuria ^#^		
Without T2DM	1.00	1.00
T2DM without albuminuria	0.84 (0.68-1.05)	1.41 (1.06-1.87) ^##^
T2DM with albuminuria	0.89 (0.54-1.46)	1.25 (0.67-2.33)

95%CI: 95% confidence interval; OR: odds ratio.

* < 7.0kg/m^2^ in men and < 5.5kg/m^2^ in women;

** Adjusted for body mass index (continuous); age; education (higher education, high school, complete elementary school, incomplete elementary school); marital status: married/cohabiting, separated/divorced, widowed, single, other); race/color (black, brown, white, yellow and Indigenous); physical activity (low, moderate or vigorous); excessive alcohol consumption (yes/no); smoking (never smoked, ex-smoker, current smoker); cardiovascular diseases (absent/present); renal dysfunction (absent/present); and use of angiotensin-converting enzyme inhibitors and/or angiotensin receptor blockers;

*** Logistic regression models;

^#^ T2DM without albuminuria: albuminuria/creatinine ratio < 30mg/g; T2DM with albuminuria: albuminuria/creatinine ratio ≥ 30mg/g;

^##^ p < 0.05.

**Table 7:** Association between type 2 diabetes mellitus (T2DM) according to the presence of albuminuria and dynapenia, with further adjustment for the use of angiotensin-converting enzyme inhibitor drugs and angiotensin receptor blockers. *Brazilian Longitudinal Study of Adult Health* (ELSA-Brasil), 2012-2014 (n = 11,619).

Dynapenia *	Crude model OR (95%CI) ***	Adjusted model ** OR (95%CI) ***
T2DM according to the presence of albuminuria ^#^		
Without T2DM	1.00	1.00
T2DM without albuminuria	1.55 (1.23-1.96) ^##^	1.32 (1.03-1.71) ^###^
T2DM with albuminuria	2.91 (1.94-4.36) ^##^	2.25 (1.45-3.49) ^##^

95%CI: 95% confidence interval; OR: odds ratio.

* Handgrip strength in men < 27kg and in women < 16kg;

** Adjusted for body mass index (continuous); age; education (higher education, high school, complete elementary school, incomplete elementary school); marital status: married/cohabiting, separated/divorced, widowed, single, other); race/color (black, brown, white, yellow and Indigenous); physical activity (low, moderate or vigorous); excessive alcohol consumption (yes/no); smoking (never smoked, ex-smoker, current smoker); cardiovascular diseases (absent/present); renal dysfunction (absent/present); and use of angiotensin-converting enzyme inhibitors and/or angiotensin receptor blockers;

*** Logistic regression models;

^#^ T2DM without albuminuria: albuminuria/creatinine ratio < 30mg/g; T2DM with albuminuria: albuminuria/creatinine ratio ≥ 30mg/g;

^##^ p < 0.001;

^###^ p < 0.05.

**Table 8:** Association between type 2 diabetes mellitus (T2DM) according to the presence of albuminuria and sarcopenia, with further adjustment for the use of angiotensin-converting enzyme inhibitor drugs and angiotensin receptor blockers. *Brazilian Longitudinal Study of Adult Health* (ELSA-Brasil), 2012-2014 (n = 11,619).

Sarcopenia (EWGSOP2)	Crude model OR (95%CI) *		Adjusted model ** OR (95%CI) *	
	Probable sarcopenia ***	Confirmed sarcopenia ^#^	Probable sarcopenia ***	Confirmed sarcopenia ^#^
T2DM according to the presence of albuminuria ^##^				
Without T2DM	1.00	1.00	1.00	1.00
T2DM without albuminuria	1.63 (1.26-2.10) ^###^	1.23 (0.70-2.16)	1.27 (0.96-1.68)	1.87 (1.01-3.44) ^###^
T2DM with albuminuria	2.72 (1.72-4.32) ^###^	3.66 (1.66-8.05) ^§^	2.04 (1.25-3.33) ^§^	4.01 (1.60-10.46) ^§^

95%CI: 95% confidence interval; EWGSOP2: European Working Group on Sarcopenia in Older People Consensus 2; OR: odds ratio.

* Multinomial regression models;

** Adjusted for body mass index (continuous); age; education (higher education, high school, complete elementary school, incomplete elementary school); marital status: married/cohabiting, separated/divorced, widowed, single, other); race/color (black, brown, white, yellow and Indigenous); physical activity (low, moderate or vigorous); excessive alcohol consumption (yes/no); smoking (never smoked, ex-smoker, current smoker); cardiovascular diseases (absent/present); renal dysfunction (absent/present); and use of angiotensin-converting enzyme inhibitors and/or angiotensin receptor blockers;

*** Handgrip strength in men < 27kg and in women < 16kg;

^#^ Simultaneous presence of dynapenia and low appendicular muscle mass;

^##^ T2DM without albuminuria: albuminuria/creatinine ratio < 30mg/g; T2DM with albuminuria: albuminuria/creatinine ratio ≥ 30mg/g;

^###^ p < 0.05;

^§^ p < 0.001.

**Table 9:** Association between type 2 diabetes mellitus (T2DM) with sarcopenia components and sarcopenia according to the European Working Group on Sarcopenia in Older People Consensus 2 (EWGSOP2), excluding individuals with cancer diagnosis. *Brazilian Longitudinal Study of Adult Health* (ELSA-Brasil), 2012-2014 (n = 11,614).

T2DM	Individual components of sarcopenia (logistic regression models)				Sarcopenia according to EWGSOP2 (multinomial regression models)			
	LAMM *		Dynapenia **		Crude model OR (95%CI)		Adjusted model *** OR (95%CI)	
	Crude model OR (95%CI)	Adjusted model *** OR (95%CI)	Crude model OR (95%CI)	Adjusted model *** OR (95%CI)	Probable sarcopenia **	Confirmed sarcopenia ^#^	Probable sarcopenia **	Confirmed sarcopenia ^#^
Absent	1.00	1.00	1.00	1.00	1.00	1.00	1.00	1.00
Present	0.86 (0.70-1.05)	1.41 (1.06-1.93) ^##^	1.65 (1.32-2.05) ^###^	1.45 (1.10-1.92) ^##^	1.69 (1.33-2.15) ^##^	1.46 (0.88-2.41)	1.34 (0.99-1.83)	2.45 (1.30-4.64) ^##^

95%CI: 95% confidence interval; LAMM: low appendicular muscle mass; OR: odds ratio.

* < 7.0kg/m^2^ in men and < 5.5kg/m^2^ in women;

** Handgrip strength in men < 27kg and in women < 16kg;

*** Adjusted for body mass index (continuous); age; education (higher education, high school, complete elementary school, incomplete elementary school); marital status: married/cohabiting, separated/divorced, widowed, single, other); race/color (black, brown, white, yellow and Indigenous); physical activity (low, moderate or vigorous); excessive alcohol consumption (yes/no); smoking (never smoked, ex-smoker, current smoker); cardiovascular diseases (absent/present), renal dysfunction (absent/present); and glycated hemoglobin (continuous);

^#^ Simultaneous presence of dynapenia and LAMM;

^##^ p < 0.05;

^###^ p < 0.001.

**Table 10:** Association between type 2 diabetes mellitus (T2DM) with sarcopenia components and sarcopenia according to the European Working Group on Sarcopenia in Older People Consensus 2 (EWGSOP2), excluding individuals with rheumatic diseases. *Brazilian Longitudinal Study of Adult Health* (ELSA-Brasil), 2012-2014 (n = 12,038).

T2DM	Components of sarcopenia (logistic regression models)				Sarcopenia according to EWGSOP2 (multinomial regression models)			
	LAMM *		Dynapenia **		Crude model OR (95%CI)		Adjusted model *** OR (95%CI)	
	Crude model OR (95%CI)	Adjusted model *** OR (95%CI)	Crude model OR (95%CI)	Adjusted model *** OR (95%CI)	Probable sarcopenia **	Confirmed sarcopenia ^#^	Probable sarcopenia **	Confirmed sarcopenia ^#^
Absent	1.00	1.00	1.00	1.00	1.00	1.00	1.00	1.00
Present	0.89 (0.73-1.09)	1.51 (1.12-2.04) ^##^	1.67 (1.35-2.07) ^###^	1.52 (1.16-1.99) ^##^	1.69 (1.33-2.13) ^###^	1.62 (1.02-2.58) ^##^	1.39 (1.03-1.88) ^##^	2.51 (1.39-4.52) ^##^

95%CI: 95% confidence interval; LAMM: low appendicular muscle mass; OR: odds ratio.

* < 7.0kg/m^2^ in men and < 5.5kg/m^2^ in women;

** Handgrip strength in men < 27kg and in women < 16kg;

*** Adjusted for body mass index (continuous); age; education (higher education, high school, complete elementary school, incomplete elementary school); marital status: married/cohabiting, separated/divorced, widowed, single, other); race/color (black, brown, white, yellow and Indigenous); physical activity (low, moderate or vigorous); excessive alcohol consumption (yes/no); smoking (never smoked, ex-smoker, current smoker); cardiovascular diseases (absent/present), renal dysfunction (absent/present); and glycated hemoglobin (continuous);

^#^ Simultaneous presence of dynapenia and LAMM;

^##^ p < 0.05;

^###^ p < 0.001.

**Table 11:** Association between type 2 diabetes mellitus (T2DM) with sarcopenia components and sarcopenia according to the European Working Group on Sarcopenia in Older People Consensus 2 (EWGSOP2), excluding individuals who used oral/parenteral corticosteroids. *Brazilian Longitudinal Study of Adult Health* (ELSA-Brasil), 2012-2014 (n = 12,020).

T2DM	Components of sarcopenia (logistic regression models)				Sarcopenia according to EWGSOP2 (multinomial regression models)			
	LAMM *		Dynapenia **		Crude model OR (95%CI)		Adjusted model *** OR (95%CI)	
	Crude model OR (95%CI)	Adjusted model *** OR (95%CI)	Crude model OR (95%CI)	Adjusted model *** OR (95%CI)	Probable sarcopenia **	Confirmed sarcopenia ^#^	Probable sarcopenia **	Confirmed sarcopenia ^#^
Absent	1.00	1.00	1.00	1.00	1.00	1.00	1.00	1.00
Present	0.88 (0.72-1.07)	1.50 (1.11-2.04) ^##^	1.68 (1.36-2.08) ^###^	1.52 (1.16-1.99) ^##^	1.71 (1.36-2.16) ^###^	1.56 (0.97-2.51)	1.41 (1.05-1.90) ^##^	2.46 (1.36-4.47) ^##^

95%CI: 95% confidence interval; LAMM: low appendicular muscle mass; OR: odds ratio.

* < 7.0kg/m^2^ in men and < 5.5kg/m^2^ in women;

** Handgrip strength in men < 27kg and in women < 16kg;

*** Adjusted for body mass index (continuous); age; education (higher education, high school, complete elementary school, incomplete elementary school); marital status: married/cohabiting, separated/divorced, widowed, single, other); race/color (black, brown, white, yellow and Indigenous); physical activity (low, moderate or vigorous); excessive alcohol consumption (yes/no); smoking (never smoked, ex-smoker, current smoker); cardiovascular diseases (absent/present), renal dysfunction (absent/present); and glycated hemoglobin (continuous);

^#^ Simultaneous presence of dynapenia and LAMM;

^##^ p < 0.05;

^###^ p < 0.001.

The Hosmer-Lemeshow tests for goodness-of-fit showed an adequate fit of the models (p > 0.05).

## Discussion

This study describes the prevalence of sarcopenia and its components, assessed according to the EWGSOP2, in a large cohort of Brazilian middle-aged and older adults living in the community. The findings support the initial hypotheses, as they show that T2DM increased the odds of LAMM, dynapenia, and probable and confirmed sarcopenia by 55%, 58%, 49%, and 153%, respectively, even after adjustments for several confounding factors were made. Regarding the duration of T2DM diagnosis, individuals with 5-9 years of diagnosis had higher odds of LAMM and confirmed sarcopenia, and those with a diagnosis duration ≥ 10 years had higher odds of dynapenia and sarcopenia (both probable and confirmed). T2DM without and especially with the presence of albuminuria was positively associated with dynapenia, whereas T2DM with albuminuria increased the odds of confirmed sarcopenia by more than 300%. Contrary to our initial expectations, glycemic control in subjects with a previous diagnosis of T2DM was not associated with any of the conditions analyzed. In addition, no interaction was observed between the explanatory variables and age.

Our results on the association of T2DM with dynapenia and sarcopenia (probable and confirmed) are consistent with previous studies conducted with different populations, regardless of the criteria employed to identify the presence of such muscle dysfunctions. For instance, in an English study on 5,290 older adults, the chance of dynapenia was found to be higher among individuals with T2DM than among those without it ^26^. A Chinese study on 1,090 community-dwelling older adults also found a positive association of T2DM with sarcopenia ^9^. Positive associations between T2DM and sarcopenia, defined according to EWGSOP2 criteria, were also found in smaller studies conducted in Spain ^27^ and Japan ^28^.

The relationship between the duration of diabetes diagnosis and sarcopenia, shown in this study, suggests that the greater the duration of exposure to the disease, the greater the chances of developing muscle impairment and sarcopenia, which is in line with the literature ^29^ ^,^ ^30^ ^,^ ^31^ ^,^ ^32^. Interestingly, in cases of confirmed sarcopenia, this relationship is observed when T2DM diagnosis has a duration of 5-10 years or ≥ 10 years, with no signs of a gradual relationship, but in cases of probable sarcopenia, this relationship is only present when T2DM diagnosis has a duration ≥ 10 years. Despite being preventable, micro and macrovascular complications are part of the T2DM pathway and are more frequent in individuals with a longer-lasting case of diabetes, a worse risk profile, and poor glycemic control ^33^. This study observed that T2DM accompanied by albuminuria doubled the chance of dynapenia and tripled the chance of confirmed sarcopenia (assessed according to the EWGSOP2), which corroborates previous results ^34^ ^,^ ^35^ ^,^ ^36^.

However, contrary to what was initially expected, we found that inadequate glycemic control (HbA1c ≥ 7%) was not associated with any of the response variables in the participants who reported having a diagnosis of T2DM. In addition, the inclusion of HbA1c as a continuous variable in the analysis that had T2DM as the explanatory variable had negligible effects on the magnitude of the associations. Some researchers have shown that poorer glycemic control, assessed with the HbA1c test, increases the long-term risk of muscle complications such as dynapenia and sarcopenia, microvascular complications, and mortality in individuals with T2DM ^16^ ^,^ ^36^ ^,^ ^37^. A cross-sectional study in Japan also demonstrated a dose-response relationship between HbA1c level and the chance of sarcopenia, regardless of anthropometric factors and the duration of diabetes ^38^. In the literature, there is debate as to whether poor glycemic control decreases muscle function or worse muscle function primarily affects glycemic control ^39^. The possibility of a bidirectional relationship is a challenging option that longitudinal studies can shed light on.

Multiple mechanisms have been proposed to explain the association of T2DM with dynapenia and sarcopenia, namely: the reduction in muscle synthesis or sensitivity to anabolic hormones such as testosterone; mitochondrial dysfunction caused by chronic hyperglycemia inducing apoptosis and atrophy or loss of muscle fibers; and the increased secretion of inflammatory cytokines (e.g., tumor necrosis factor-α and interleukin-6) decreasing the action of glucose transporters and generating impairment in energy metabolism ^8^ ^,^ ^9^ ^,^ ^16^ ^,^ ^30^ ^,^ ^31^ ^,^ ^32^ ^,^ ^33^ ^,^ ^34^ ^,^ ^35^ ^,^ ^40^. Peripheral insulin resistance is one of the main mechanisms leading to diabetes-related impairments in muscle function. It is known that skeletal muscle is responsible for much of the postprandial glucose uptake ^40^ and that insulin acts on muscle function by facilitating and increasing the transport of glucose and amino acid to muscle cells, increasing cellular protein synthesis and storage, as well as glycogen in the muscles and triglycerides in fat cells, stimulating protein synthesis in muscles, and decreasing protein catabolism ^32^. Insulin resistance, which is characteristic of T2DM, alters glucose utilization by skeletal muscle, thus negatively affecting protein synthesis, causing proteolytic activity, and consequently leading to the loss of muscle mass and/or strength ^32^ ^,^ ^33^. Insulin resistance can increase the gene expression of myostatin, an inhibitor of skeletal muscle growth ^32^ ^,^ ^33^. Nevertheless, the loss of muscle mass and strength leads to decreases in glucose transport, which may worsen insulin resistance - this process results in a negative vicious cycle between insulin resistance and muscle dysfunction ^33^. Lastly, fat infiltration in skeletal muscle due to obesity, which happens more commonly in individuals with diabetes than in those without it, aggravates insulin resistance and deteriorates glucose metabolism, consequently compromising muscle function ^36^.

Even though the dose-response association between the duration of T2DM and increased prevalence of sarcopenia, which was found in this study, suggests a longitudinal association, the cross-sectional design of our analysis hampers any causal evaluation between T2DM and sarcopenia. In addition, a self-reported diagnosis of T2DM, which may be less accurate than objective laboratorial parameters, was one of the criteria used to ascertain the exposure of interest. However, the presence of non-differential misclassifications would contribute to bias the estimated OR towards the null in this study. The cut-off points for dynapenia and LAMM used in this study were validated in older populations of North American and European countries, which means we might have under- or super-estimated the presence of muscle dysfunction in younger populations from different regions of the world, such as ours. Longitudinal studies with outcomes known to be associated with sarcopenia in younger and older Brazilians are needed to validate the proposed EWGSOP2 cut-off points. Furthermore, although BIA is an accepted and accessible method for estimating body composition, it has limitations for assessing skeletal muscle mass, especially in individuals with obesity ^37^. The estimation of body composition made by BIA is based on the values of resistance and inductive reactance derived from the electric current generated by the equipment that travels through the body during the analysis ^41^. However, while body fat is estimated directly by BIA, muscle mass is estimated by prediction equations ^42^. Such equations are calibrated based on the lean mass obtained by DEXA in a specific population chosen according to the device used and the population studied ^37^ ^,^ ^43^ ^,^ ^44^ ^,^ ^45^. Lastly, although HGS is a good indicator of overall muscle strength, it shows a moderate correlation with strength in other body parts ^7^ ^,^ ^46^. A three-year follow-up study with 1,840 subjects aged 70-79 showed that the 50% faster decline in knee extensor strength in older adults with T2DM was not explained by a greater loss of leg muscle mass ^47^. Nonetheless, in the study, the changes in arm strength and muscle quality did not differ between those with and without diabetes ^47^.

Progress in clinical research shows that the force generated by a muscle is not directly proportional to its size and to the amount of muscle fiber, and that the decline in muscle strength happens at a faster rate than the loss of muscle mass - which corroborates the change in the algorithm proposed by the EWGSOP2 ^1^ ^,^ ^48^ ^,^ ^49^. Among the strengths of the present work, we highlight the use of EWGSOP2 definitions of sarcopenia and its components, the large sample size, and the inclusion of middle-aged and older adults from three regions of Brazil. In addition, the present analysis considered the influence of numerous potential confounders, and our results remained the same even in sensitivity analyses that excluded comorbidities strongly associated with sarcopenia (e.g., cancer and rheumatic diseases).

It is noteworthy that the presence of dynapenia and sarcopenia in individuals with T2DM is associated with a significant increase in adverse events that negatively impact quality of life ^36^ ^,^ ^50^. This emphasizes the importance of monitoring musculoskeletal functions in individuals with the disease in order to detect early changes. Furthermore, the use of different operational definitions of the components of sarcopenia obscures our understanding of these relationships. In order to promote early diagnosis, it is necessary to standardize and validate the definition and cut-off points of dynapenia and sarcopenia in different and younger populations. The early screening of muscle strength and mass has been identified as an effective strategy for minimizing this deterioration and promoting the maintenance of quality of life. Thus, further research with longitudinal follow-up and evaluation of outcomes associated with muscle dysfunction is important to better understand the relationship between T2DM and sarcopenia and its components, and would allow for the planning of earlier interventions and for the prevention of potential negative outcomes of the disease.
